# Evaluation and DFT Analysis of In Vitro Anticancer Activity of *Consolida orientalis*, *Smyrnium rotundifolium*, and *Euphorbia virgata* Plant Extracts in Colorectal Cancer

**DOI:** 10.3390/ph18070943

**Published:** 2025-06-22

**Authors:** Eda Sönmez Gürer, Zuhal Tunçbilek, Cemile Zontul, Ahu Kutlay, Amrendra Kumar, Gaurav Jhaa

**Affiliations:** 1 Department of Pharmacognosy, Faculty of Pharmacy, Sivas Cumhuriyet University, Sivas 58140, Türkiye; 2 Department of Chemistry and Chemical Processing Technologies Services, Yıldızeli Vocational School, Sivas 58140, Türkiye; zuhaltuncbilek@cumhuriyet.edu.tr (Z.T.); cemilezontul@cumhuriyet.edu.tr (C.Z.); 3 Department of Forestry, Koyulhisar Vocational School, Sivas Cumhuriyet University, Sivas 58140, Türkiye; akutlay@cumhuriyet.edu.tr; 4 Department of Bioinformatics, NIMS Institute of Allied Medical Science and Technology, NIMS University, Jaipur 303121, Rajasthan, India; amrendra.kumar@nimsuniversity.org; 5 Department of Chemical Sciences, Indian Institute of Science Education and Research (IISER), Mohali 140306, Punjab, India; gauravjhaa@iisermohali.ac.in

**Keywords:** colorectal cancer, *Consolida orientalis*, DFT, *Euphorbia virgata*, *Smyrnium rotundifolium*, molecular docking

## Abstract

**Background**: Colon cancer is one of the leading causes of cancer-related deaths today. Crucial research continues for the ideal chemotherapy. In this context, natural compounds of plant origin play an important role in the development of new anticancer drugs. **Methods**: In this study, the effects of *Consolida orientalis* ethanol extract (flower parts), *Smyrnium rotundifolium* ethanol extract (aerial parts), and *Euphorbia virgata* ethanol extract (aerial parts) samples on HT-29 (human colorectal adenocarcinoma cell line) and healthy CCD-18Co (human normal colon fibroblast cell line) were investigated for the first time in the literature by applying 3-(4,5-dimethylthiazol-2-yl)-2,5-diphenyl tetrazolium bromide (MTT) test within the scope of in vitro cytotoxicity analysis. **Results**: As a result of the study, it was observed that all plant extracts were most effective at 72 h. *S. rotundifolium* ethanol extract (aerial parts) was found to be the most effective on the HT-29 cell line. Both the higher cell viability of *C. orientalis* in healthy cells applied to it compared to *S. rotundifolium* and its effectiveness on colon cancer cell lines make *C. orientalis* more advantageous. **Conclusions**: When evaluating the efficacy of extracts on cancer cells, the load on healthy cells should be taken into account. Therefore, *C. orientalis* ethanol extract (flower parts) was found to have the potential to be a chemotherapeutic agent against colon cancer. Chemical reactivities of the dominant components of bioactive components were analyzed via Conceptual Density Functional Theory-based calculations. The power of the interactions with EGFR kinase of these compounds is checked via Molecular Docking Calculations. It was noted that Chlorogenic acid, which is the most reactive bioactive component, has a stronger binding to the mentioned enzyme.

## 1. Introduction

The leading cause of death worldwide is cardiovascular disease, followed by cancer. Cancer is one of the most common diseases and has a high mortality rate, making it one of the most important health problems in the world. Cancer is a pathological disease that begins with the uncontrolled growth of cells and can occur as a result of genetic factors and ongoing life processes [1,2].

Colon cancer is the second most commonly diagnosed cancer in women and the third most commonly diagnosed cancer in men, and according to World Health Organization data, it is the third most common cause of cancer-related deaths [2]. The rates in developed countries are much higher than in other countries. It is known that colorectal cancer cases worldwide will reach 2.5 million new cases in 2035 [3]. This alarming increase shows us that colon cancer will continue to be a serious public health problem in the future. Although genetic factors are the main cause of the disease’s pathogenesis, an unhealthy diet, tobacco and alcohol use, and inflammatory bowel disease are also involved [4]. Since the symptoms of the disease appear in the late stages, early diagnosis contributes significantly to reducing the risk of death. Diagnostic methods include colonoscopy and liquid biopsy. The treatment of the disease is performed with methods such as surgery, radiotherapy, and chemotherapy, and the increasing mortality rate shows us that the currently used methods are unsuccessful. There are many anticancer drugs available on the market that are frequently used in chemotherapy. However, due to drug-related toxic effects, low selectivity and the development of drug resistance, the development of new generation anticancer drugs with low toxicity and high selectivity has become extremely important [1,5]. For this purpose, the development of synthetic, semi-synthetic or natural pharmaceutical raw materials has become quite popular.

Natural compounds of plant origin play an important role in the development of new anticancer drugs. It is known that medicinal plants have antioxidant properties, anti-inflammatory properties, anti-mutagenic effects, and anti-angiogenic effects thanks to the many phytochemicals they contain (such as flavonoids, polyphenol compounds, caffeic acid, catechins, saponins, polysaccharides, triterpenoids, alkaloids, glycosides, phenols, quercetin, luteolin, kaempferol, rosmarinic acid, emodin, eugenol); as a result, they reduce the side effects of chemotherapy and reduce the rate of cancer recurrence and spread to other organs. For this purpose, traditional natural medicines are used all over the world to prevent and treat cancer [6,7]. Compounds with confirmed anticancer activity to date include vinblastine, paclitaxel, podophyllotoxin, camptothecin, curcumin, and cannabinoids. Two drug structures have been identified by the National Cancer Institute (NCI) for their biological activities by both in vitro and in vivo methods—paclitaxel and camptothecin [7,8].

In the present study, we aimed to investigate the anticancer effects of three medicinal plants, *Consolida orientalis*, *Smyrnium rotundifolium*, and *Euphorbia virgata. C. orientalis* is native to the southern/eastern parts of Europe and is abundant in southeastern Hungary [9]. The aim of this study was to evaluate the in vitro cytotoxic activity of the ethanol extract of *C. orientalis* collected from Mazandaran in northern Iran using the human cervical carcinoma cell line HeLa. The results of the study demonstrated the anticancer potential of the ethanol extract of the plant in the HeLa cell line. This result demonstrated the presence of cytotoxic compounds in ethanol extracts of *C. orientalis* [10]. In another study conducted to determine the cytotoxic effect of *C. orientalis* flower extracts, it was determined that the plant extracts did not show any cytotoxic effect on the WI-38 human fibroblast cell line even at a concentration of 5 mg/100 μL [11]. Smyrnium, which belongs to the Apiaceae family and has 38 species in the world distribution, is represented in Turkey by six taxa, including *S. rotundifolium*. It is a biennial herbaceous plant that grows in stony places, bushes, and forest edges [12]. In a study conducted with leaf extracts of the species *S. rotundifolium*, the total phenol content was found to be 157.3 mg GAE/g extract and exhibited low antioxidant activity [13]. It contains kaempferol (5 mg) and its 3-/9-D-galactoside (10 mg), kaempferol 3-methyl ether 7-jS-D-glucoside (15 mg), and a kaempferol 3-diglucoside (15 mg) flavonoids [14]. The Euphorbia genus is the genus with the most species in the Euphorbiaceae family. Euphorbia species contain latex. This genus, which has nearly 2000 species, is usually seen in Africa or Madagascar. There are 91 *Euphorbia* species growing in Turkey. In Turkey, its flowers are used in the treatment of eczema [15]. Studies have shown that *Euphorbia* species have cytotoxic, antitumor, antibacterial, anti-inflammatory, and anti-HIV activities [16]. Flavonoid derivatives isolated from *E. virgata* are quercetin-3-O-glucoside, kaempferol, kaempferol-3-O-glucoside, kaempferol-3-rutinoside, and rutin [17].

## 2. Results and Discussion

### 2.1. Extract Yields

After the extraction process was carried out with three different plants, the yield of the obtained extract was weighed and calculated as g/100 g dry sample. The highest extract yield was obtained in *S. rotundifolium* ethanol extract (aerial parts) with 10.6%, while the lowest yield was determined as 3.3% in *C. orientalis* ethanol extract (flower parts). The yield of *E. virgata* ethanol extract (aerial parts) was determined as 8.5%.

### 2.2. Cytotoxicity Assay

Cytotoxic analysis of *C. orientalis* ethanol extract (flower parts), *S. rotundifolium* ethanol extract (aerial parts), *E. virgata* ethanol extract (aerial parts) samples, and docetaxel drug was performed on HT-29 cancer cells and healthy CCD-18Co cells. The chemotherapeutic agent docetaxel is included in the study as a reference for comparison. In this context, the obtained IC_50_ data were compared (Table 1). The doses of *C. orientalis* ethanol extract (flower parts), *S. rotundifolium* ethanol extract (aerial parts), and *E. virgata* ethanol extract (aerial parts) ranged from 0.5 to 250 µg/mL, while docetaxel doses ranged from 1 to 100 µM.

**Figure 1:**
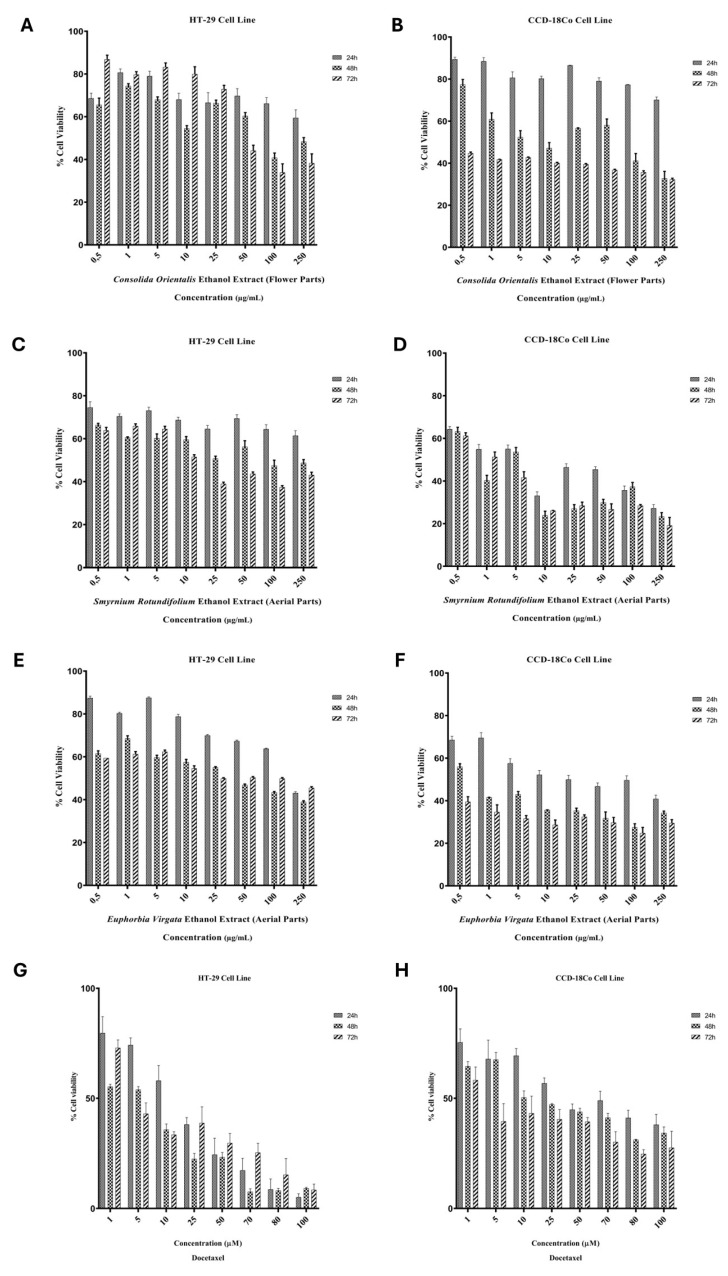
The effects of *C. orientalis* (flower part), *S. rotundifolium* (aerial part), *E. virgata* (aerial part) ethanol extracts, and docetaxel treatment on cell viability in HT-29 and CCD-18Co cell lines, as determined by MTT assay. The left panel shows results for HT-29 cells, while the right panel presents data for CCD-18Co cells. (**A**): *C. orientalis* (flower part) extract applied to HT-29 cell line; (**B**): *C. orientalis* (flower part) extract applied to CCD-18Co cell line; (**C**): *S. rotundifolium* (aerial part) extract applied to HT-29 cell line; (**D**): *S. rotundifolium* (aerial part) extract applied to CCD-18Co cell line; (**E**): *E. virgata* (aerial part) extract applied to HT-29 cell line; (**F**): *E. virgata* (aerial part) extract applied to CCD-18Co cell line; (**G**): Docetaxel treatment applied to HT-29 cell line; (**H**): Docetaxel treatment applied to CCD-18Co cell line.

It was observed that all plant extracts were most effective at 72 h. *S. rotundifolium* ethanol extract (aerial parts) was found to be the most effective on the HT-29 cell line. *E. virgata* ethanol ext. (aerial parts) was found to be the most effective on the CCD-18Co cell line, but it should be kept in mind that this extract negatively affects the viability of healthy cells. It is observed that the cytotoxic dose of *C. orientalis* ethanol extract (flower parts) is less effective than the cytotoxic dose of *S. rotundifolium* ethanol extract (aerial parts). However, the lower effect of *C. orientalis* ethanol extract on cell viability of healthy cells compared to *S. rotundifolium* ethanol extract indicates that it is more advantageous. When evaluating the effectiveness of extracts on cancer cells, the load on healthy cells should be taken into account. Therefore, *C. orientalis* ethanol extract (flower parts) can be accepted as an effective extract for colorectal adenocarcinoma. The sample with the highest cytotoxic effect on HT-29 cells was determined to be *S. rotundifolium* ethanol extract (aerial parts). *E. virgata* ethanol extract (aerial parts) was effective on HT-29 cells, but similar to *S. rotundifolium* ethanol extract (aerial parts) extract, it was noted that although it was effective on colorectal adenocarcinoma, its cytotoxic effect on healthy cells was higher. Cell viability graphs were obtained including the extracts applied to both cell lines (Figure 1).

**Figure 2:**
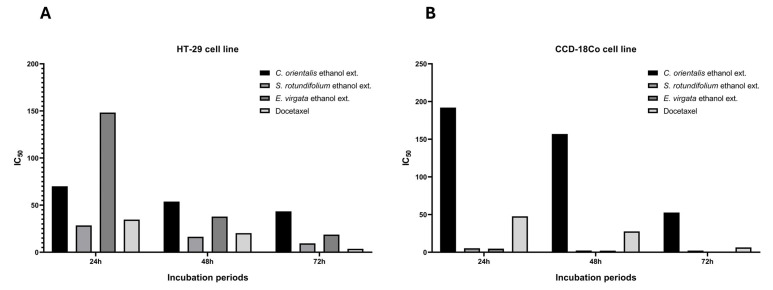
Graph representing the effects of plant extracts and docetaxel on (**A**) HT-29 and (**B**) CCD-18Co cell lines following 24, 48, and 72 h of incubation, based on IC_50_ values.

Two-way ANOVA was performed to evaluate the effects of incubation time on the samples applied to HT-29 and CCD-18Co cell lines. Accordingly, in the HT-29 cell line, a statistically significant difference was observed among all plant extracts and docetaxel at each incubation time point (24, 48, and 72 h) (**** *p* < 0.0001). In the CCD-18Co cell line, the same analysis revealed significant differences at all time points between *C. orientalis* (flower part) and *S. rotundifolium* (aerial part), *C. orientalis* (flower part) and *E. virgata* (aerial part), and *C. orientalis* (flower part) and docetaxel. These findings suggest that the relatively higher IC_50_ value of *C. orientalis* (flower part) in the healthy CCD-18Co cell line resulted in significant differences when compared to the other treatment groups (*p* < 0.05) (Figure 2).

### 2.3. Biotic Compound and Target Proteins Identifications

A total of 14 biotics compounds were found in the IMPPAT database of *C. orientalis* (Table 2). We found further target proteins in Swiss Target Predictions (STP) databases, where 200 proteins and enzymes have been identified. Additionally, 196 colorectal cancer proteins and enzymes were found in IMIM databases, in which one EGFR kinase protein has an overlap with three flower phytochemicals of *C. orientalis,* as well as being colorectal cancer-related.

### 2.4. Bonding Energy Analysis Between Ligands and Proteins

**Figure 3:**
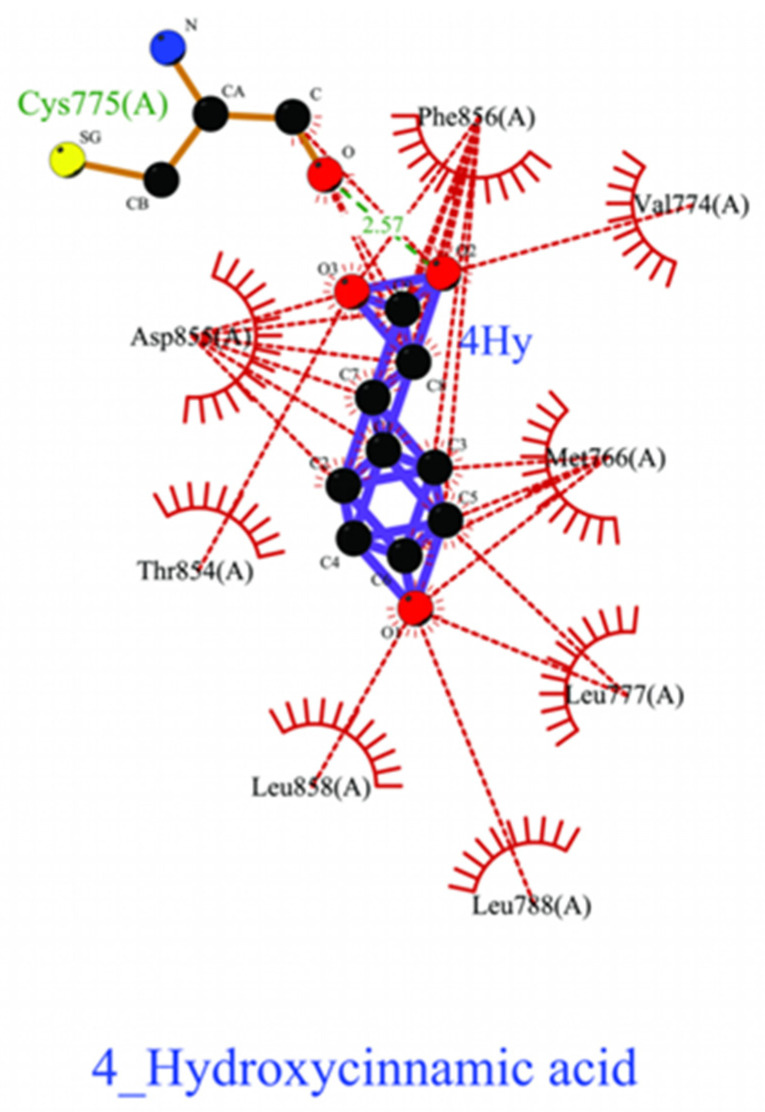
Two-dimensional depiction of the molecular interaction between 4_Hydroxycinnamic acid and colorectal cancer protein EGFR kinase. The green line represents hydrogen bonds, while the red line indicates hydrophobic interactions.

**Figure 4:**
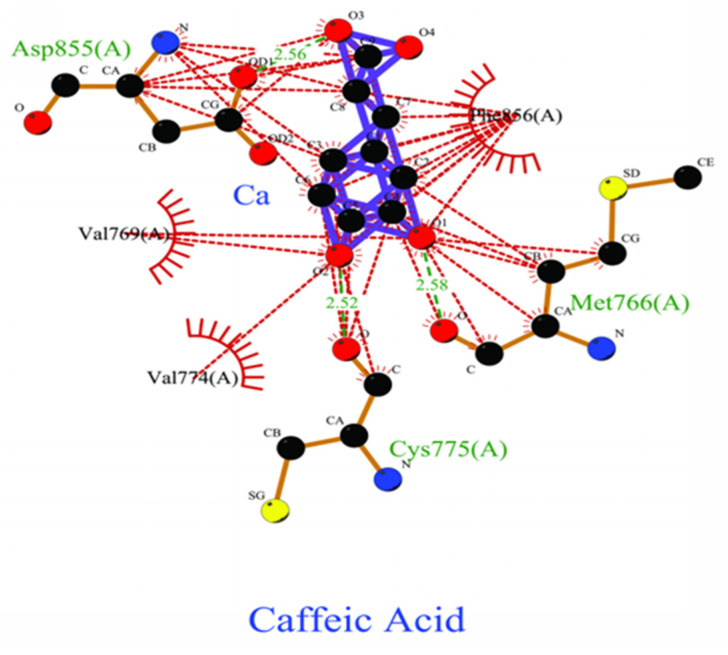
Two-dimensional depiction of the molecular interaction between Caffeic acid and colorectal cancer protein EGFR kinase. The green line represents hydrogen bonds, while the red line indicates hydrophobic interactions.

**Figure 5:**
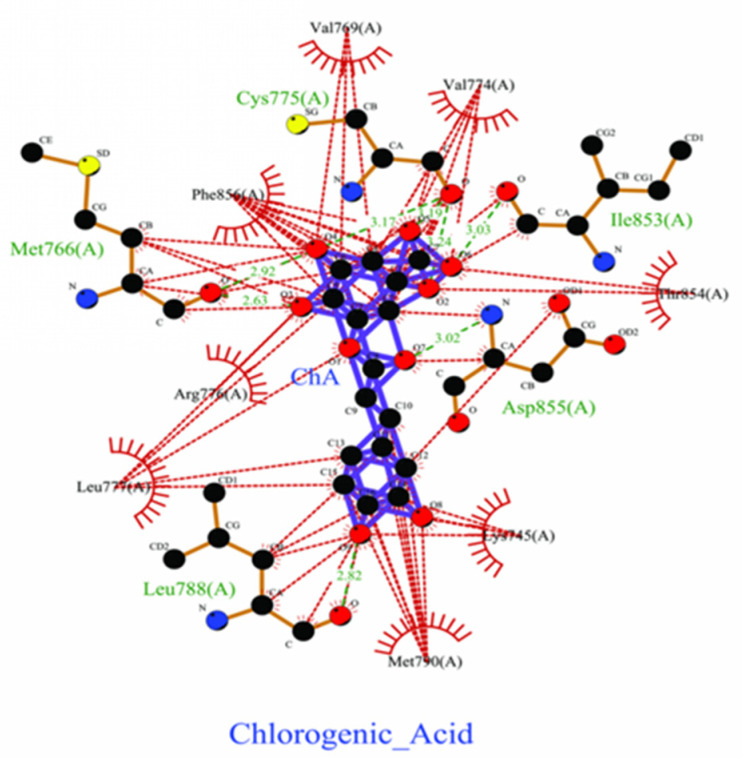
Two-dimensional depiction of the molecular interaction between Chlorogenic acid and d colorectal cancer protein EGFR kinase. The green line represents hydrogen bonds, while the red line indicates hydrophobic interactions.

Three lead-like phytochemical compounds, namely 4_Hydroxycinnamic Acid (PubChem ID: 637542), Caffeic Acid (PubChem ID: 689043), and Chlorogenic Acid (PubChem ID: 1794427) were identified. 4_Hydroxycinnamic Acid exhibited a ΔG value of −6.38 kcal/mol and engaged with nine residues through one hydrogen bond and eight residues with hydrophobic interactions (Figure 3). Similarly, Chlorogenic acid showed a ΔG value of −6.34 kcal/mol, interacting with six residues through Vdw, three hydrogen bonds, and three hydrophobic interactions (Figure 4). Finally, Chlorogenic acid demonstrated ΔG values of −13.28 kcal/mol, and it interacted with five hydrogen bonds; the other eight had hydrophobic interactions with EGFR kinase (PDB ID: 7JXM) (Figure 5).

### 2.5. Evaluation of Docked Complex Proteins Stability Through MD Simulation

**Figure 6:**
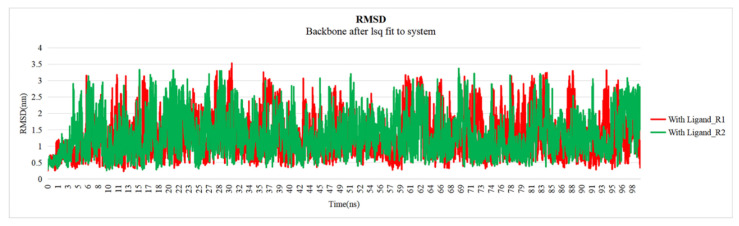
RMSD plot of the chlorogenic acid–EGFR protein complex, where R1 and R2 represent two independent replicate molecular dynamics (MD) simulations over time.

The interaction between Chlorogenic acid and the target EGFR kinase (PDB ID: 7JXM) protein was found to have five hydrogen bonds. Further molecular dynamics simulation studies exhibited hydrogen bonding during docking. The stability of these complexes was assessed through a 100 ns molecular dynamics simulation using the GROMACS package, with RMSD and RMSF. The evaluation of the stability of docked complexes is primarily dependent on root-mean-square deviation (RMSD) parameters. The mean RMSD values for chlorogenic acid bound with EGFR were found to be 0.6 nm. The RMSD plot revealed that the protein–chlorogenic acid complex exhibited minimal deviation, suggesting a higher level of stability, while without (Figure 6).

Additional analyses revealed that, beyond the total binding energy, van der Waals and electrostatic interactions significantly contribute to the stability of the complexes. For the chlorogenic acid–EGFR protein complex, the electrostatic energy was approximately −17,640.29 kcal/mol, and the van der Waals energy was −2031.53 kcal/mol, as reported in Table S1A. On average, the van der Waals (VDWAALS) and electrostatic (EEL) energy components were found to be −2009.89 kcal/mol and −17,545.7 kcal/mol, respectively (Table S1B), indicating their substantial role in complex stabilization.

### 2.6. Root Mean Square Fluctuation of EGRF Proteins

**Figure 7:**
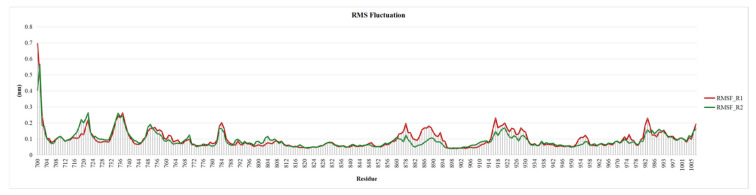
RMSF Plot of two replicate R1 and R2 with Chlorogenic acid complex EGFR proteins.

Root Mean Square Fluctuation (RMSF) is a critical tool for assessing the dynamic flexibility of docked complexes. The average RMSF values for the docked complexes of EGRF (0.54 nm) provide valuable insights into the structural fluctuations that occur during molecular dynamics simulations. Notably, lower RMSF values were found in hydrogen bond interactions Iis853, ASP 855, etc., while the hydrophobic interaction site experienced more fluctuations (Figure 7).

**Figure 8:**
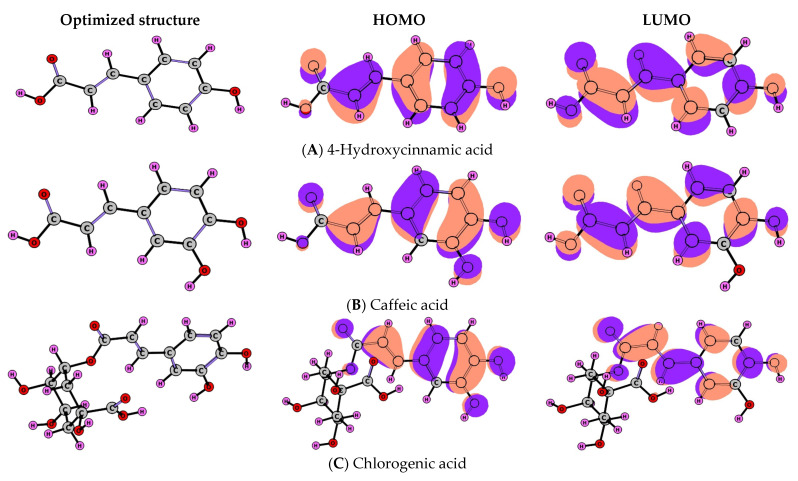
Optimized geometry and Frontier MOs of (**A**) 4-Hydroxycinnamic acid, (**B**) Caffeic acid, and (**C**) Chlorogenic acid in solvent phase (water) at B3LYP/6-311+G** with GD3.

**Figure 9:**
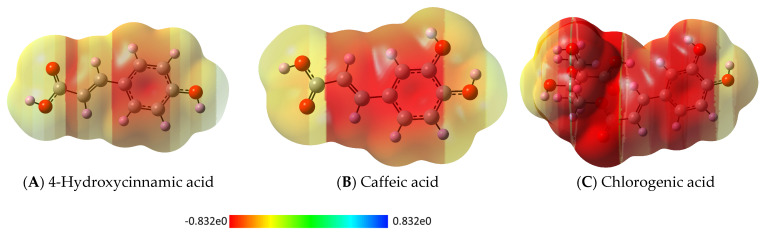
ESP maps of (**A**) 4-Hydroxycinnamic acid, (**B**) Caffeic acid, and (**C**) Chlorogenic acid in solvent phase (water).

Conceptual Density Functional Theory-based parameters are widely preferred for explaining the nature and power of the interactions between chemical systems. Visually, Figure 8 shows the optimized structure, with HOMO and LUMO images of 4-Hydroxycinnamic acid, Caffeic acid, and Chlorogenic acid, which are the dominant molecular components of the extract with high biological activity among the studied extracts. Figure 9 presents the ESP (Electrostatic Potential) maps of the same molecules. Red regions in ESP indicate a negative ESP (electron excess or loosely bound electrons), while blue regions indicate a positive ESP (electron deficiency). A high electrostatic potential indicates the relative absence of electrons, and a low electrostatic potential indicates an abundance of electrons. In Table 3, calculated quantum chemical descriptors of the studied molecular systems are given. According to bonding energy analysis made via the Molecular Docking approach [18,19], the strongest interaction with EGFR kinase is shown for Chlorogenic acid.

Chemical hardness [20] and electrophilicity index [21] have been closely related parameters to the chemical reactivity of the molecular systems via Maximum Hardness [22] and Minimum Electrophilicity Principles [23]. Chemical hardness, showing the resistance against the polarization of the atoms, ions, and molecules, is a good measure of stability. According to the Maximum Hardness Principle, “there seems to be a rule of nature that molecules arrange themselves so as to be as hard as possible.” In light of the calculated chemical hardness values given in the related table, it can be said that among the molecules studied, the hardest and most stable is 4-Hydroxycinnamic acid, while the most reactive and soft is Chlorogenic acid. Minimum Electrophilicity Principle implies that the electrophilicity index is minimized in stable states like polarizability. The Minimum Electrophilicity Principle, like the Maximum Hardness Principle, presents the stability ranking of the studied molecules as 4-Hydroxycinnamic acid> Caffeic acid> Chlorogenic acid. As seen from the results, Chlorogenic acid, which has lower hardness and higher electrophilicity index, has a stronger bond with the studied biological system. On the other hand, stable and hard chemical systems interacted less with the biological system under study.

Overall, the global reactivity descriptors—such as chemical hardness, softness, electronegativity, and electrophilicity index—were calculated to evaluate the reactive behavior of the dominant molecules. These parameters are strongly correlated with biological activity, as molecules with optimal values often exhibit stronger interactions with biological targets. Moreover, the frontier molecular orbital (HOMO–LUMO) gap analysis was used to estimate the kinetic stability and possible electron donation/acceptance tendencies of the compounds. For instance, compounds with a lower HOMO-LUMO gap are typically more reactive and may more effectively interact with macromolecular targets involved in oxidative stress and cancer pathways. These theoretical insights support the antioxidant and anticancer potential demonstrated in the in vitro assays, thus establishing a computational basis for their observed bioactivity. A molecule with a larger value of softness has a small energy separation and shows high reactivity as it can easily donate electrons to the acceptor part. Thus, a molecule with smaller hardness values and higher softness values suggests larger chemical reactivity.

## 3. Materials and Methods

### 3.1. Plant Material

The *C. orientalis*, *S. rotundifolium*, and *E. virgata* plants used in the study were collected from the area of Sivas İmaret village at 39°41′40” N, 37°02′25” E coordinates, at an altitude of 1400 m, in June–July 2023. The taxonomic identification of the collected samples was made by Anadolu University, Faculty of Pharmacy, Prof. Dr. Yavuz Bülent KÖSE. A sample of each collected plant was labeled with a herbarium number (*C. orientalis*: 16195, *S. rotundifolium*: 16196, *E. virgata:* 16197) and recorded and stored in the Anadolu University Faculty of Pharmacy Herbarium.

### 3.2. Preparation of Plant Extract

In our study, plant extracts prepared from *C. orientalis* flower parts, *S. rotundifolium*, and *E. virgata* aerial parts were used. The samples were first washed with tap water and then with distilled water, then dried on blotting papers, ground in a grinder, then 100 g were taken and 1000 mL of ethanol (Merck, Darmstadt, Germany) was added. It was kept at room temperature in a shaker at 150 rpm for 24 h. At the end of the extraction process, the extract was filtered through filter paper, then the solvent was removed in a rotary evaporator at 40 °C. The obtained extracts were taken into dark glass bottles and stored at −20 °C to be used in experimental procedures [24].

### 3.3. Cell Culture

HT-29 (Human colorectal adenocarcinoma cell line) and CCD-18Co (human normal colon fibroblast cell line) cells were purchased from an American Type Culture Collection (ATCC), United States. Roswell Park Memorial Institute (RPMI) 1640 Medium was used for HT-29 cells. Dulbecco’s Modified Eagle Medium (DMEM) was used for CCD-18Co cells. The medium was prepared with 10% fetal bovine serum (FBS) and 1% penicillin–streptomycin. The cells were grown at 5% CO_2_ and 37 °C. When the cells reached 80–90% fullness, they were passaged to other flasks.

### 3.4. In Vitro Cytotoxicity Assay

Within the scope of in vitro cytotoxicity analysis, 3-(4,5-dimethylthiazol-2-yl)-2,5-diphenyl tetrazolium bromide (MTT) assay was applied [25]. For this purpose, firstly, cells with a density of 1 × 10^5^ cells/well were seeded into 96 well plates. The cells were incubated under appropriate conditions for one day. Then, *C. orientalis* ethanol extract (flower parts), *S. rotundifolium* ethanol extract (aerial parts), and *E. virgata* ethanol extract (aerial parts) samples were prepared at varying concentrations including 250, 100, 50, 25, 10, 5, 1, and 0.5 µg/mL, and applied to HT-29 and CCD-18Co cell lines. The cells to which plant extracts were applied at varying concentrations were incubated for 24, 48, and 72 h, respectively, and cell viability was analyzed at the end of these periods. Each set of experiments was performed in triplicate. IC_50_ values were obtained using GraphPad Prism v8 application and cytotoxicity graphs were created. The obtained data were compared with each other.

### 3.5. Molecular Docking Analysis

#### 3.5.1. Bioactive Compound Identifications

The bioactive compound of *C. orientalis* will be collected from the accomplished work, as well as from experimentally proven research published in reputed journals and the Indian Medicinal Plants, Phytochemistry and Therapeutics (IMPPAT) database [26], and then converted and downloaded in SMILES format.

#### 3.5.2. Target Protein Identifications Linked to Bioactive or Hypertensive

The Swiss Target Prediction (STP) tools will be used to identify target proteins with filtered bioactivity by entering their SMILES with homo sapiens. In contrast, colorectal cancer-related proteins will be collected from publicly available databases, OMIM [27] and DisGeNET [28]. Further, the Venn diagram will display the target combined targets among bioactive and colorectal cancer proteins.

#### 3.5.3. Preparation of Ligand, Target Protein, and Stability Check

The structure of bioactive compounds will be retrieved from PubChem databases in .sdf file format of 4_Hydroxycinnamic Acid (PubChem ID: 637542), Caffeic Acid (PubChem Id: 689043), and Chlorogenic Acid (PubChem ID: 1794427). The 3D structure of colorectal cancer protein will be downloaded from the Protein Databank of EGFR kinase (PDB ID: 7JXM) [29]. Furthermore, docking was performed using AutoDock 4 with MGLTools version 1.5.7, employing the default parameters. The grid center was set at coordinates (24.907, –29.423, 83.493), focusing on the active binding site of the protein. The protein–ligand complex visualized by the LigPlot + (V2) (https://www.ebi.ac.uk/thornton-srv/software/LigPlus/, accessed on 4 May 2025) and PyMol (Education use only) with high docking score and their interaction with key residues will be further considered for the classical molecular studies.

#### 3.5.4. MD Simulation of EGFR Kinase Docked Complexes with Top Ligand

Molecular dynamics simulations were carried out on complexes formed by the EGFR kinase (PDB ID: 7JXM) protein, with the top Chlorogenic acid exhibiting the lowest ΔG and at least five hydrogen bonds. The GROMACS package [30] (v2024.3) was used to extract the selected drugs from the docked complexes using the gmx grep module. Topologies for the target protein were generated using the pdb2gmx module, and the CHARMM General Force Field (CGenFF) server was employed to assign the necessary force field to the selected ligand hits. The system underwent 25,000,000-step minimization using the steepest descent algorithm. The system temperature gradually increased from 0 to 300 K during a 100 ps equilibration phase at constant NVT and NPT. Finally, a 100 ns MD simulation of the protein–ligand system was conducted under identical conditions at 1 bar and a temperature of 300 K. Analysis of the simulation trajectories was performed using the gmx rms and gmx rmsf modules to visualize RMSD and RMSF.

### 3.6. Details of DFT Calculations

Quantum chemical calculations based on the Density Functional Theory (DFT) were performed on gas-phase molecules using the Gaussian 16 Revision C.02 computational framework. The B3LYP hybrid functional was employed with the 6-311++G * basis set, supplemented by Grimme’s GD3 empirical dispersion correction to account for weak interactions. All of the calculations were performed in solvent media (water) using the SMD solvation approach. The stability of the stationary points was evaluated through vibrational frequency analysis, which confirmed the nature of the critical points as the local minimum due to the absence of imaginary frequencies. Additionally, wave function stability tests were conducted for all molecular structures to ensure the reliability and robustness of the electronic solutions. The Conceptual Density Functional Theory (CDFT) presents the following relations to compute important reactivity descriptors such as chemical potential (*µ*), electronegativity (*χ*), hardness (*η*), first (*ω*_1_), and second (*ω*_2_) electrophilicity indexes for atomic and molecular systems [31,32,33].

$$\mu =-\chi ={\left[\frac{\partial E}{\partial N}\right]}_{v(r)}=-[\frac{I+A}{2}],$$

$$\eta ={\left[\frac{\partial \mu }{\partial N}\right]}_{v(r)}={\left[\frac{\partial^{2}E}{\partial N^{2}}\right]}_{v(r)}=I-A,$$

$$\omega_{1}=\chi^{2\;}/2\eta =\mu^{2}/2\eta ={\left(I+A\right)}^{2}/8(I-A),$$

$$\omega_{2}=I.A/(I-A),$$

here E, N, I, and A represent the total electronic energy, total number of the electrons, ionization energy, and electron affinity of the studied chemical system, respectively.

For approximately calculating the ionization energy and electron affinity of the studied molecules, we considered the Koopmans Theorem (KT) [34], giving the following relations:

$$I=-E_{HOMO},$$

$$A=-E_{LUMO}$$

where E_HOMO_ and E_LUMO_ represent the energies of HOMO and LUMO orbital of the molecular chemical systems, respectively.

## 4. Conclusions

There are very few comprehensive studies in the literature about the anticancer activity of ethanol extracts of *C. orientalis* (flower parts), *S. rotundifolium* (aerial parts), and *E. virgata* (aerial parts). In the experimental part of the study, the effects of the plants’ ethanol extracts on HT-29 (human colorectal adenocarcinoma cell line) and healthy CCD-18Co (human normal colon fibroblast cell line) were evaluated by means of a 3-(4,5-dimethylthiazol-2-yl)-2,5-diphenyl tetrazolium bromide (MTT) test. The experiments proved that the activity of *C. orientalis* extract against colorectal cancer is much higher than that of other extracts. GC-MS analysis revealed several bioactive phytoconstituents, among which the dominant compounds 4-Hydroxycinnamic acid, Caffeic acid, and Chlorogenic acid were further analyzed using Conceptual DFT calculations. Theoretical descriptors such as chemical hardness, electrophilicity, and HOMO–LUMO energy gaps provided insights into their chemical reactivity and potential mechanisms of biological action. The Maximum Hardness and Minimum Polarizability Principles implied that the most reactive component with the lower chemical hardness of *C. orientalis* is Chlorogenic acid. The performed Molecular Docking calculations showed that Chlorogenic acid interacts more powerfully with EGFR kinase than Caffeic acid and 4-Hydroxycinnamic acid.

## Figures and Tables

**Table 1:** IC_50_ values of *C. orientalis* ethanol extract (flower parts), *S. rotundifolium* ethanol extract (aerial parts), and *E. virgata* ethanol extract (aerial parts) samples and docetaxel applied to HT-29 and CCD-18Co cells at doses of 250–0.5 µg/mL.

Plant Extracts	HT-29			CCD-18Co		
	24 h	48 h	72 h	24 h	48 h	72 h
*C. orientalis* ethanol ext.	70.00	53.82	43.43	191.90	156.90	52.67
*S. rotundifolium* ethanol ext.	28.48	16.47	9.44	5.34	2.35	2.25
*E. virgata* ethanol ext.	148.30	37.85	18.75	4.80	2.13	0.32
Docetaxel	34.68	20.31	3.70	47.67	27.69	6.44

**Table 2:** Biotics compound.

No	Plant Name	Plant Parts	Phytochemical Name
1	*C. orientalis*	flower	2,4-Dihydroxy-2H-1,4-benzoxazin-3(4H)-one
2	*C. orientalis*	flower	2-Benzoxazolinone
3	*C. orientalis*	flower	4-Hydroxybenzoic acid
4	*C. orientalis*	flower	Chlorogenic acid
5	*C. orientalis*	flower	Caffeic acid
6	*C. orientalis*	flower	4-Hydroxycinnamic acid
7	*C. orientalis*	seed	Ajadinine
8	*C. orientalis*	seed	Ajacine
9	*C. orientalis*	seed	Unii-DW4U480C61
10	*C. orientalis*	seed	(1S,2R,3R,4S,5R,6S,8R,9S,10S,13S,16S,17R,18S)-11-ethyl-13-(hydroxymethyl)-4,6,18-trimethoxy-11-azahexacyclo [7.7.2.12,5.01,10.03,8.013,17]nonadecane-8,9,16-triol
11	*C. orientalis*	seed	Delsoline
12	*C. orientalis*	seed	(1S,2R,3R,4S,5S,6S,8R,9S,10S,13S,16S,17R,18S)-11-ethyl-6,18-dimethoxy-13-(methoxymethyl)-11-azahexacyclo [7.7.2.12,5.01,10.03,8.013,17]nonadecane-4,8,9,16-tetrol
13	*C. orientalis*	NA	(1S,2R,3R,4S,5R,6S,8R,9S,10S,13S,16S,17R,18S)-11-ethyl-13-(hydroxymethyl)-4,6,18-trimethoxy-11-azahexacyclo [7.7.2.12,5.01,10.03,8.013,17]nonadecane-8,9,16-triol
14	*C. orientalis*	NA	18-hydroxy-14-O-methylgadesine

**Table 3:** Calculated quantum chemical descriptors for the studied bioactive compounds in solvent phase (water).

	E_HOMO_ (eV)	E_LUMO_ (eV)	χ	η	ω_1_	ω_2_	Dipole Moment (Debye)	α (a.u)
4-Hydroxycinnamic acid	−6.235	−2.135	4.185	4.100	2.136	3.247	5.56	202.811
Caffeic acid	−6.112	−2.159	4.136	3.953	2.164	3.338	5.07	211.760
Chlorogenic acid	−6.054	−2.088	4.071	3.966	2.089	3.187	4.38	350.107

## Data Availability

Data are contained within the article and Supplementary Materials.

## Supplementary Materials

The following supporting information can be downloaded at: https://www.mdpi.com/article/10.3390/ph18070943/s1. Table S1. A: Details of MMPB/SA calculation from Chlorogenic acid and d colorectal cancer protein EGFR kinase. B: MMPB/SA calculation from Chlorogenic acid and d colorectal cancer protein EGFR kinase.
